# Translation, cross-cultural adaptation and initial psychometric evaluation of the Indonesian PSPSQ 2.0 in community pharmacies and public primary healthcare centres

**DOI:** 10.1016/j.rcsop.2026.100843

**Published:** 2026-09-14

**Authors:** Irianti Bahana Maulida Reyaan, Tjokorde Istri Armina Padmasawitri, Sherly Meilianti, I Ketut Adnyana, Lia Amalia

**Affiliations:** a Department of Pharmacology-Clinical Pharmacy, School of Pharmacy, Institut Teknologi Bandung, Bandung, Indonesia; b School of Pharmacy, Applied Sciences and Public Health, Robert Gordon University, Scotland, United Kingdom; c Department of Pharmacy Practice, Faculty of Pharmacy, Universitas Airlangga, Surabaya, Indonesia

**Keywords:** Pharmacist, Patient satisfaction, Community pharmacy, Primary healthcare settings, Cross-cultural adaptation

## Abstract

**Background:**

Patient satisfaction is a key indicator of healthcare quality and patient-reported experience. However, a culturally adapted and psychometrically evaluated Indonesian version of the Patient Satisfaction with Pharmacist Services Questionnaire (PSPSQ 2.0) has not been reported.

**Objectives:**

This study aimed to translate, culturally adapt, and evaluate the initial psychometric properties of the Indonesian version of the PSPSQ 2.0 among patients receiving pharmacist services in community pharmacies (*apotek*) and public primary healthcare centres (*Puskesmas*).

**Methods:**

Patients were purposively recruited via an anonymous online questionnaire. The instrument underwent forward–backward translation, expert review, content validity assessment, and cognitive debriefing. Structural validity was assessed using exploratory factor analysis with a polychoric correlation matrix, MINRES extraction, and oblimin rotation. Factor retention was assessed using eigenvalues, scree-plot inspection, and Horn's parallel analysis. Internal consistency was evaluated using Cronbach's alpha and McDonald's omega.

**Results:**

A total of 200 participants were included (58% female; mean age 29.85 ± 9.07 years). I-CVI values ranged from 0.80 to 1.00, with an overall S-CVI/Ave of 0.975. The KMO was 0.920, with significant Bartlett's test (χ^2^ = 3826.157, df = 190, *p* < 0.001). EFA provided preliminary evidence for a three-factor structure comprising Quality of Care (QOC), Interpersonal Relationship (IPR), and Overall (OV), supported by converging factor-retention criteria and interpretable factor-loading patterns. Cronbach's alpha and McDonald's omega ranged from 0.867 to 0.928 and 0.870 to 0.931, respectively, across the three domains. For the total scale, α was 0.960 and ω was 0.968.

**Conclusion:**

The Indonesian PSPSQ 2.0 demonstrated preliminary evidence of structural validity and internal consistency for assessing patient satisfaction in community and primary care settings. Further studies are needed to confirm its factor structure and applicability across broader and more diverse populations.

## Highlights

• Indonesian version of the PSPSQ 2.0 was translated and culturally adapted.
• Exploratory factor analysis supported three factors: quality of care, interpersonal relationships, and overall satisfaction.
• Indonesian PSPSQ 2.0 demonstrated good structural validity and internal consistency.
• Instrument can assess patient satisfaction with pharmacist services in community pharmacies and primary healthcare centres.
• Preliminary evidence supports standardized evaluation of pharmaceutical services and future research in Indonesia.

## 1 Introduction

Patient satisfaction is widely regarded as a key indicator of healthcare quality, reflecting individuals' perceptions and experiences with healthcare services and settings. It is shaped by multiple factors, including quality of care, provider communication, accessibility, costs, organizational aspects, and health outcomes.^1^ Within pharmaceutical care, patient satisfaction is particularly relevant because it captures the experiential dimension of pharmacist–patient interactions, including counseling quality, responsiveness, and perceived trust.^2^ However, satisfaction is a multidimensional construct influenced by expectations, cultural norms, and healthcare delivery models,^3^, ^4^ meaning that accurate measurement requires instruments that are both psychometrically sound and contextually appropriate. Generic satisfaction tools may not adequately capture specific aspects of pharmacist services, such as medication counseling, drug information provision, and follow-up care. Therefore, validated pharmacy-specific instruments are needed to ensure that key dimensions of pharmaceutical care are appropriately represented.^5^

In parallel, pharmacists' roles have shifted from product-oriented dispensing toward patient-centred services, including medication management, health promotion, and chronic disease care.^6^ In Indonesia, these services are provided through both community pharmacies (*apotek*) and public primary healthcare centres (*Puskesmas*), which differ in organizational roles and healthcare integration. In Indonesia, *Puskesmas* may participate in government-supported chronic disease programmes such as *Prolanis*, in which pharmacists can contribute to medication education and monitoring, whereas such programme-based integration is less inherent to community pharmacy practice,^7^ whereas community pharmacies (*apotek*) primarily operate as community-based pharmacy settings, providing services that may include prescription assessment, counseling, medication monitoring, and homecare. These differences in service context and the integration of pharmacists into broader care programmes may influence pharmacist–patient interactions and patient satisfaction.^8^ Internationally, the scope of pharmacist services also varies across healthcare settings and systems. For example, in Aotearoa New Zealand, some pharmacists working within collaborative primary healthcare teams may undertake extended clinical roles, including prescribing within a defined scope of practice.^9^

One established instrument for assessing patient satisfaction with pharmacist services is the Patient Satisfaction with Pharmacist Services Questionnaire (PSPSQ 2.0), which measures quality of care, interpersonal relationship, and overall satisfaction.^10^ Compared with other healthcare satisfaction measures, such as the the Patient Satisfaction Questionnaire (PSQ) provides a generic assessment of satisfaction with medical care, whereas the Perceived Service Quality Scale (PSQS) specifically assesses perceived service quality in community pharmacies, including technical quality, relationship quality, environmental quality, non-prescription services, and health outcomes. Thus, the PSPSQ 2.0 was selected because it specifically focuses on patient satisfaction with pharmacist-provided care, making it closely aligned with the objective of evaluating contemporary pharmacist services and pharmacist–patient interactions.^10^, ^11^, ^12^

Despite its strengths, the use of PSPSQ 2.0 in different cultural and healthcare contexts requires careful translation and cross-cultural adaptation to ensure semantic, conceptual, and technical equivalence.^13^, ^14^ Standard guidelines for instrument adaptation recommend a structured, multi-step process to ensure linguistic and conceptual equivalence across settings. This typically includes forward translation, synthesis of translations, back translation, harmonization, pre-testing, field testing, and subsequent psychometric validation and analysis of measurement properties.^15^ This systematic approach is essential to preserve the instrument's measurement properties while ensuring cultural relevance and contextual appropriateness for the target population. Such procedures commonly include assessment of internal consistency and structural validity, providing preliminary evidence that the adapted instrument measures the intended domains in the target population.^16^

In Indonesia, the coexistence of community pharmacies (*apotek*) and *Puskesmas* as important points of access to primary healthcare creates a need for a patient satisfaction measure that is appropriate to these local service contexts.^8^, ^17^ However, validated pharmacy-specific instruments remain limited, with many studies relying on non-standardized measures that restrict comparability. To date, no study has translated, culturally adapted, and psychometrically evaluated the PSPSQ 2.0 across both settings in Indonesia. This gap underscores the need for a culturally appropriate Indonesian measure to support standardized assessment of patient satisfaction with pharmacist services in primary care and inform quality improvement and future research.

Therefore, this study aimed to translate, cross-culturally adapt, and evaluate the initial psychometric properties of the Indonesian version of the PSPSQ 2.0 among patients receiving pharmacist services in community pharmacies (*Apotek*) and public primary healthcare centres (*Puskesmas)*. The resulting Indonesian adaptation is intended to provide a culturally appropriate measure of patient satisfaction for use in pharmacy practice and research in Indonesia.

## 2 Methods

### 2.1 Study design and settings

This methodological cross-sectional study translated, culturally adapted, and evaluated the initial psychometric properties of the Indonesian version of the Patient Satisfaction with Pharmacist Services Questionnaire (PSPSQ 2.0). The translation and cross-cultural adaptation process was guided by established recommendations for patient-reported outcome measures, including the World Health Organization guidance and the ISPOR Good Practice Guidelines, covering forward translation, reconciliation, back translation, expert review, pre-testing/cognitive debriefing, and psychometric evaluation.^18^

This study was conducted in Indonesia, a Southeast Asian archipelagic country comprising more than 17,000 islands and administratively divided into 38 provinces. Data were collected from the Indonesian population across 35 provinces. The data collection was carried out over a defined study period in a confidential setting, where participants were informed about the study objectives prior to providing consent.

### 2.2 Study population, inclusion and exclusion criteria

A purposive sampling technique was employed to recruit participants who met the study criteria. Eligible participants were adults aged ≥18 years who reported receiving services directly from a pharmacist in a community pharmacy (*apotek*) or public primary healthcare centre (*Puskesmas*) within the previous six months. Pharmacist services in these settings include clinical pharmacy activities such as prescription screening and dispensing, provision of drug information, counseling, medication therapy monitoring, and monitoring of adverse drug reactions. Additional services may include home pharmacy care in community pharmacies and patient visits in inpatient Puskesmas. Participants were instructed to answer the questionnaire based on their most recent pharmacist service encounter in the selected setting.

Eligibility was screened through a multi-step procedure before access to the main questionnaire. Participants were asked to report the type of healthcare facility visited (community pharmacy or public primary healthcare centre), frequency of visits, and the time since their most recent visit (categorized as ≤1 month, >1–3 months, or > 3–6 months), as well as their current health condition. These responses were used to assess consistency with the predefined eligibility criteria and to identify responses that were incomplete or inconsistent. Participants who did not meet the eligibility criteria or had missing PSPSQ 2.0 responses were excluded. Only complete responses were included in the final analysis. All participants provided electronic informed consent. The minimum sample size was estimated using the common rule of thumb of 5–10 participants per item for factor analysis.^19^ As the PSPSQ 2.0 consists of 20 items, the recommended range was 100–200 participants. The final analytic sample of 200 participants met the upper end of this recommended range.

### 2.3 Data collection procedures

**Fig. 1:**
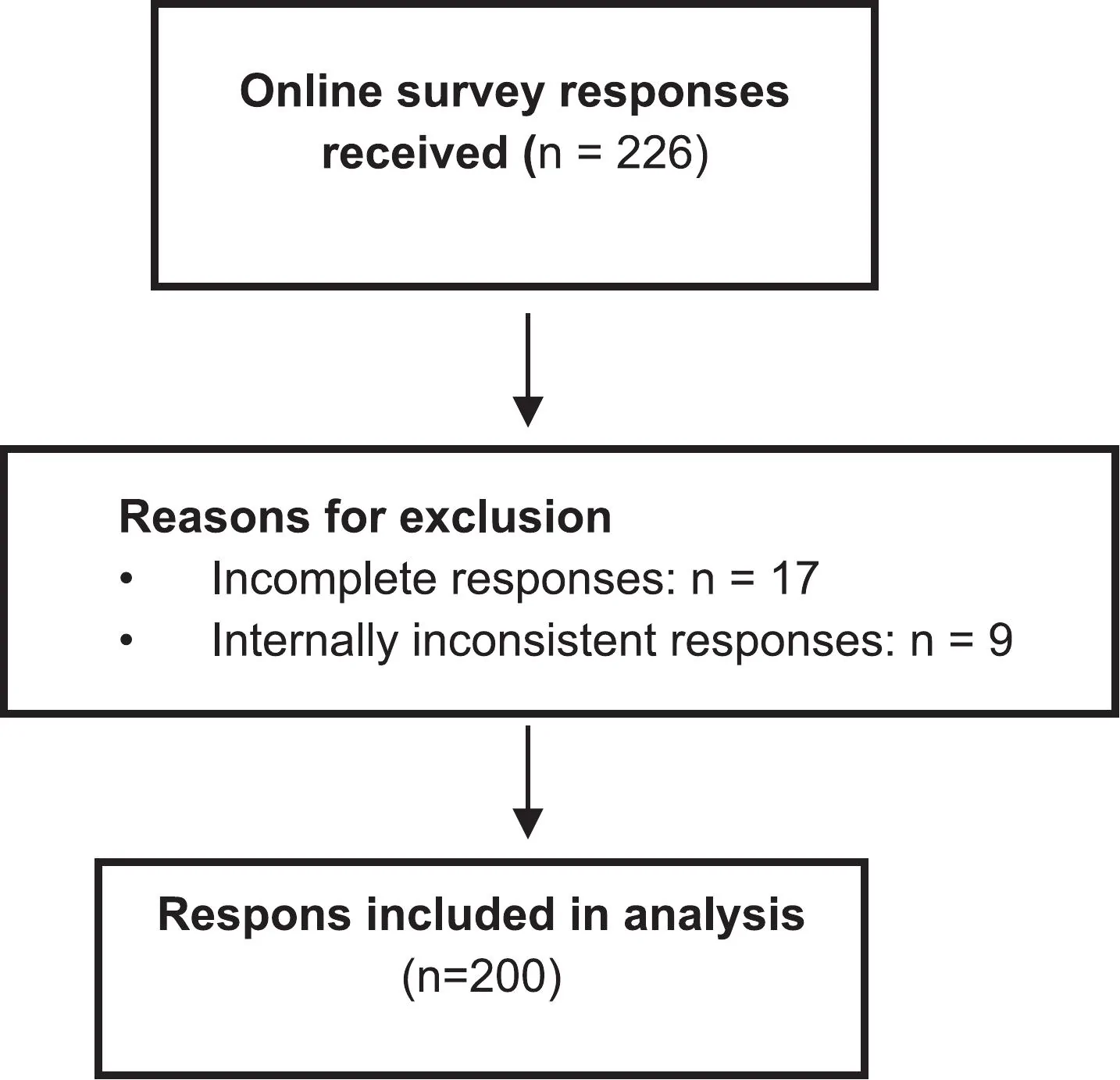
Participant flow and data-quality screening for the PSPSQ 2.0 online survey.

Data were collected through an anonymous online questionnaire distributed through professional organisations, academic networks, and community contacts. These channels were used to circulate the survey link to members of the public who met the eligibility criteria and had received pharmacist services. The first page of questionnaire provided study information, eligibility criteria, and an electronic informed consent statement. Participants who met all eligibility criteria proceeded to demographic questions and the PSPSQ 2.0 items. The questionnaire required approximately 15–20 min to complete, and no incentives were provided. Before statistical analysis, responses were screened according to predefined data-quality criteria. Incomplete responses were defined as responses in which one or more required questionnaire items were left unanswered. Non-differentiated response patterns were defined a priori as selecting the same response category across all 20 PSPSQ 2.0 items and were considered indicative of potentially inattentive responding. The survey did not collect direct personal identifiers such as names, email addresses, or telephone numbers. Submission IP addresses and timestamps were available from the survey platform and were used as part of the data-quality screening process. Potential duplicate submissions were screened based on IP address and submission timestamp information, followed by comparison of available demographic characteristics and item-level response patterns. A shared IP address or closely spaced timestamp was not considered sufficient evidence of duplicate participation, because multiple respondents could access the survey through the same network. Accordingly, duplicate participation could not be definitively confirmed or ruled out in this anonymous survey. No response was excluded as a duplicate solely on the basis of this screening process. Responses meeting the predefined exclusion criteria were removed before statistical analysis. A total of 226 responses were received, of which 17 incomplete and 9 internally inconsistent responses were excluded (see Fig. 1).

### 2.4 Research instruments

Original PSPSQ 2.0.

The original English version of the PSPSQ 2.0, developed by Sakharkar et al., comprises 20 items across three latent domains: quality of care (10 items), patient–pharmacist relationship (6 items), and overall satisfaction (4 items) (see supplementary materials Table S1).^10^ Each item is rated on a four-point Likert-type scale (1 = strongly disagree to 4 = strongly agree), enabling quantification of satisfaction levels across domains. A demographic questionnaire was additionally captured, including age, sex, highest educational attainment, employment status, marital status, type of healthcare facility visited, frequency of healthcare visits, timing of their most recent visit, geographic region, and self-reported health condition. The procedures for translation, cross-cultural adaptation, and psychometric evaluation (including validity and internal consistency testing) were conducted in accordance with established methodological frameworks and are described in detail in the subsequent subsections.

### 2.5 Translation, cross-cultural adaptation, and content validity

The PSPSQ 2.0 was translated and culturally adapted into the Indonesian language following formal authorization obtained from the original developer, Sakharkar, via email.^17^ The adaptation process followed a structured multi-stage approach involving sworn translators and an expert panel. A sworn translator independently translated the original English version of the PSPSQ 2.0 into Indonesian (forward translation). The translated version was reviewed by the research team and reconciled through discussion to produce a consensus Indonesian version. This version was subsequently back-translated into English by a second independent sworn translator who was blinded to the original instrument. The original, translated, and back-translated versions were then compared to evaluate semantic and conceptual equivalence, with any discrepancies resolved through consensus among the translators and research team.

A multidisciplinary panel of ten experts with backgrounds in community pharmacy, public primary healthcare settings, academia, and health policy reviewed the translated version for semantic, idiomatic, experiential, and conceptual equivalence, as well as item relevance and clarity (see supplementary materials Table S2). Content validity was assessed using a 4-point relevance scale and quantified using the item-level content validity index (I-CVI) and scale-level content validity index (S-CVI), with values of ≥0.78 and ≥ 0.90, respectively considered acceptable.^20^ Pilot testing with cognitive debriefing was conducted with 10 respondents from the target population, focusing on item clarity, comprehensibility, interpretation, and cultural relevance.

### 2.6 Descriptive analysis

Participant characteristics and other categorical variables were summarized using frequencies and percentages. The online questionnaire was configured to require responses to all PSPSQ 2.0 items before submission. Therefore, no missing data were present for the instrument items, and complete-case analysis was performed.

### 2.7 Comparative analysis by practice setting

The distributions of the QOC, IPR, and OV domain component scores were compared between community pharmacies and public primary healthcare centres (Puskesmas). Shapiro–Wilk tests indicated significant deviations from normality for all domain scores in both practice settings (all *p* < 0.001). Therefore, two-sided Mann–Whitney *U* tests were used to compare the distributions of observed domain scores between the two settings (α = 0.05).^21^ Scores were summarized as medians and interquartile ranges (IQRs). This analysis was conducted as an additional comparison of observed domain scores and was not intended to assess measurement equivalence, measurement invariance, or cross-setting validity. Comparability of the instrument across practice settings may be further examined in future studies using multigroup confirmatory factor analysis (MGCFA) with measurement invariance testing or differential item functioning (DIF) analysis.^22^

### 2.8 PSPSQ 2.0 scoring and domain-score calculation

The PSPSQ 2.0 was scored according to its original three-domain structure: Quality of Care (QOC; 10 items), Interpersonal Relationship (IPR; 6 items), and Overall (OV; 4 items). Domain scores were calculated as the mean of the corresponding item scores, with a theoretical range of 1–4. The total score was calculated as the mean across all 20 items, also ranging from 1 to 4. Higher scores indicated greater satisfaction. No items required reverse coding.

### 2.9 Structural validation and internal consistency analysis

*Structural Validity.*

Structural validity was assessed using exploratory factor analysis (EFA). Because the PSPSQ 2.0 uses a four-point Likert scale, items were treated as ordinal variables. Sampling adequacy was assessed using the Kaiser–Meyer–Olkin (KMO) measure, with values ≥0.60 considered acceptable, and Bartlett's test of sphericity, with *p* < 0.05 indicating suitability for factor analysis.^23^

A polychoric correlation matrix was used, and factor extraction was performed using minimum residual (MINRES) extraction with oblimin rotation. The number of factors was assessed using Kaiser's criterion (eigenvalue >1.0), scree-plot inspection, and Horn's parallel analysis. Parallel analysis used the same polychoric correlation matrix and MINRES extraction method as the EFA.24., 25 One-, two-, and three-factor solutions were compared based on factor retention criteria and relative model fit, with the overall factor solution further considered in relation to factor interpretability and the original PSPSQ 2.0 conceptual domains.^26^ Item retention was guided by factor loadings, with loadings of 0.30–0.40 considered minimally acceptable and ≥ 0.50 considered practically significant.^27^ Cross-loading was assessed using the 0.40–0.30–0.20 rule. An item was classified as having substantive cross-loading when an alternative factor loading was ≥0.30 and the difference between the primary and highest alternative loading was <0.20.^28^ The final solution was selected based on the overall psychometric evidence and theoretical interpretability. All statistical analyses were conducted using IBM SPSS® Statistics for Windows, version 31.0 (IBM Corp., Armonk, NY, USA) and R version 4.5.3 (R Foundation for Statistical Computing, Vienna, Austria), with the *psych* package version 2.6.5.

### 2.10 Internal consistency

Internal consistency reliability was assessed for the total scale and each PSPSQ 2.0 domain using Cronbach's alpha (α) and McDonald's omega (ω). Values ≥0.70 were considered indicative of acceptable internal consistency for each domain.^29^ Item-level diagnostics, including corrected item–total correlations, squared multiple correlations, and changes in Cronbach's alpha and McDonald's omega following item deletion, were also examined to assess item performance. Ninety-five percent confidence intervals (CIs) were calculated for both α and ω. Domain-level coefficients were considered the primary internal consistency estimates, whereas coefficients for the total scale were reported for completeness.

### 2.11 Ethical considerations

This study received ethical approval from the Health Research Ethics Committee of Universitas Padjadjaran (Komite Etik Penelitian Kesehatan UNPAD) under approval number No:180/UN6.KEP/EC/2025, dated 03-03-2025. The study was conducted in accordance with the principles of the Declaration of Helsinki.^30^ Electronic informed consent was obtained from all participants before data collection. Permission to translate, culturally adapt, and use the PSPSQ 2.0 was obtained from the instrument copyright holder/developer before the study commenced.^10^ A non-commercial licensing agreement was in place before use of the instrument.

## 3 Results

### 3.1 Socio-demographic characteristics

A total of 200 participants were included in the analysis. The mean age was 29.85 ± 9.07 years, and 116 participants were female (58.0%). Participants were recruited from community pharmacies (*n* = 91, 45.5%) and *Puskesmas* (*n* = 109, 54.5%). Most participants reported 1–2 healthcare visits per month (75.5%). All participants had received pharmacist services within the previous 6 months, with 41.0% reporting their most recent service within the past month. The most commonly reported health conditions were acute respiratory infections, minor ailments, and hypertension. Over half of the participants held a bachelor's degree (51.5%). Participants represented various regions of Indonesia, with Java accounting for the largest proportion (see Table 1).

**Table 1:** Demographic Characteristics of Respondents (*n* = 200).

Variables	Categories	Frequency	Percentage (%)
Age (years)	18–30	125	62.5
	31–45	59	29.5
	46–60	16	8.0
Gender	Male	84	42.0
	Female	116	58.0
Type of healthcare facilities	Community pharmacy	91	45.5
	Primary healthcare centres (*Puskesmas*)	109	54.5
Frequency of healthcare visits/month	1–2 times	151	75.5
	3–4 times	40	20.0
	5–6 times	5	2.5
	>6 times	4	2.0
Time since most recent pharmacist service	≤1 month	82	41.0
	>1–3 months	69	34.5
	>3–6 months	49	24.5
Self-reported health condition	Acute Respiratory Infections (e.g., common cold, cough)	44	22.0
	Minor Ailments (e.g., headache, mild fever, minor pain)	36	18.0
	Hypertension	32	16.0
	Gastrointestinal Disorders (e.g., gastritis, GERD, dyspepsia)	26	13.0
	Diabetes Mellitus	24	12.0
	Musculoskeletal Disorders (e.g., myalgia, low back pain)	16	8.0
	Skin Conditions (e.g., dermatitis, fungal infections)	10	5.0
	Allergic Conditions (e.g., rhinitis, urticaria)	6	3.0
	Dyslipidemia	6	3.0
Highest Educational Attainment	Senior high school	42	21.0
	Diploma (D3)	14	7.0
	Bachelor's Degree	103	51.5
	Master's Degree	36	18.0
	Doctoral Degree	5	2.5
Marital Status	Married	96	48.0
	Not married	104	52.0
Island Regions	Sumatra	33	16.5
	Java	83	41.5
	Bali and Nusa Tenggara	23	11.5
	Kalimantan	25	12.5
	Sulawesi	20	10.0
	Maluku & Papua	16	8.0

### 3.2 Comparative analysis by practice setting

No statistically significant differences were observed in the distributions of the QOC, IPR, and OV domain scores between community pharmacies and *Puskesmas* (all *p* > 0.05; see Supplementary materials Table S3). These findings indicate that the observed domain scores did not differ statistically between the two practice settings. However, the absence of statistically significant differences should not be interpreted as evidence of measurement equivalence, measurement invariance, or cross-setting validity.

### 3.3 Translation, cross-cultural adaptation, and content validity

The translation and cultural adaptation process resulted in the final Indonesian version of the PSPSQ 2.0 (see supplementary materials Table S6). Comparison of the original, translated, and back-translated versions identified no major conceptual discrepancies. Expert review supported the semantic, idiomatic, experiential, and conceptual equivalence of the translated items. Content validity was assessed using a 4-point relevance scale, with I-CVI values ranging from 0.80 to 1.00 and an overall S-CVI/Ave of 0.975 (see supplementary materials Table S4). Cognitive debriefing identified minor wording issues in Q2 and Q5, which were refined while preserving the original meaning, whereas Q14 was retained without wording changes following clarification. All 20 items were retained in the final Indonesian version (see supplementary materials Table S5 and supplementary materials Table S6). Overall, all 20 items were considered understandable, relevant, and culturally appropriate, with no reported difficulties in interpreting the items or response options.

### 3.4 PSPSQ 2.0 scoring

The observed mean scores were similar across the three PSPSQ 2.0 domains, with QOC and IPR showing comparable scores and OV showing a slightly lower mean score. The overall PSPSQ 2.0 mean score was 3.420, with all domain and total scores falling within the theoretical range of 1–4 (see Table 2).

**Table 2:** PSPSQ 2.0 domain scoring and theoretical score ranges.

Domain	Items	Scoring method	Theoretical range	Observed mean
Quality of Care (QOC)	QOC1–QOC10	Mean	1–4	3.431
Interpersonal Relationship (IPR)	IPR1–IPR6	Mean	1–4	3.430
Overall (OV)	OV1–OV4	Mean	1–4	3.379
Total PSPSQ 2.0	20 items	Mean	1–4	3.420

### 3.5 Intercorrelation analysis

The intercorrelation analysis (see Table 3) based on the polychoric correlation matrix, showed moderate to strong and statistically significant correlations among the three factors (*r* = 0.539–0.708, *p* < 0.001). None of the inter-factor correlations exceeded 0.90, suggesting that the factors were related but did not exhibit excessive overlap or redundancy.^31^ At the item level, polychoric correlations ranged from 0.390 to 0.839 (see supplementary materials Table S7), with no correlation exceeding 0.90. Overall, these findings support the relatedness of the three factors while indicating sufficient empirical distinctiveness to retain them as separate dimensions.

**Table 3:** Inter-construct correlations.

	Factor 1	Factor 2	Factor 3
Factor 1	1.000	0.708	0.639
Factor 2	0.708	1.000	0.539
Factor 3	0.639	0.539	1.000

All correlations are significant at *p* < 0.001.

### 3.6 Structural validity

The 20-item dataset was suitable for factor analysis (KMO = 0.920; Bartlett's test: χ^2^ = 3826.157, df = 190, *p* < 0.001). The scree plot (see supplementary materials Fig. S1) showed an inflection after the third factor, supporting a three-factor solution. This finding was consistent with the Kaiser criterion, which indicated three factors with eigenvalues greater than 1 (see Table 4), and Horn's parallel analysis, which also indicated a three-factor solution (see supplementary materials Fig.S2). Comparison of the alternative factor solutions showed that the three-factor solution provided the most favourable relative fit across the solutions examined (see supplementary materials Table S8). Considering these statistical criteria together with factor loading patterns (see Table 5), factor interpretability, and correspondence with the original PSPSQ 2.0 conceptual domains, a three-factor solution was retained. Accordingly, the three-factor solution was interpreted as preliminary evidence of structural validity rather than as a confirmed factor structure.

**Table 4:** Eigenvalues and percentage of variance (*n* = 200).

Factor	Eigenvalue	% of variance	Cumulative %
1	12.280	61.398	61.398
2	1.187	5.934	67.332
3	1.077	5.383	72.715
4	0.760	3.800	76.515
5	0.617	3.085	79.600
6	0.514	2.570	82.170
7	0.482	2.408	84.578
8	0.476	2.382	86.960
9	0.389	1.946	88.906
10	0.348	1.740	90.646
11	0.322	1.608	92.255
12	0.293	1.467	93.722
13	0.245	1.226	94.948
14	0.222	1.108	96.056
15	0.194	0.968	97.023
16	0.168	0.840	97.863
17	0.149	0.747	98.611
18	0.124	0.622	99.233
19	0.087	0.437	99.670
20	0.066	0.330	100.000

**Table 5:** Results of the EFA Analysis in the Indonesian PSPSQ 2.0 Domain (*n* = 200).

Item	Factor 1	Factor 2	Factor 3	Communalities
Q1	0.530	0.328	0.094	0.670
Q2	0.621	0.254	−0.179	0.772
Q3	0.891	−0.127	−0.050	0.817
Q4	0.761	0.043	0.097	0.784
Q5	0.648	0.218	0.022	0.725
Q6	0.771	0.041	0.150	0.746
Q7	0.695	0.160	0.140	0.775
Q8	0.561	0.351	0.183	0.790
Q9	0.520	0.305	0.071	0.765
Q10	0.574	0.154	0.358	0.782
Q11	0.199	0.649	0.032	0.909
Q12	0.026	0.654	0.273	0.727
Q13	−0.143	0.981	−0.048	0.774
Q14	0.109	0.726	0.014	0.717
Q15	−0.022	0.623	0.319	0.802
Q16	0.333	0.604	−0.261	0.804
Q17	−0.142	0.281	0.671	0.875
Q18	0.163	0.023	0.740	0.784
Q19	0.025	0.171	0.638	0.703
Q20	0.104	0.211	0.456	0.571

As shown in Table 4, the initial eigenvalues indicated that the first factor accounted for the largest proportion of variance (61.398%), followed by the second (5.934%) and third factors (5.383%). After extraction using minimum residual (MINRES) factoring, the three retained factors accounted for 68.126% of the total variance, with Factors 1, 2, and 3 accounting for 32.258%, 27.345%, and 8.523%, respectively.

All items had primary factor loadings ≥0.456 in the pattern matrix (see Table 5). Six items (Q1, Q8, Q9, Q10, Q15, and Q16) had alternative factor loadings ≥0.30. However, the differences between their primary and highest alternative loadings were ≥ 0.20 (Δ = 0.202–0.304); therefore, none met the predefined criterion for substantive cross-loading based on the 0.40–0.30–0.20 criterion.

### 3.7 Internal consistency

Internal consistency was high across all three PSPSQ 2.0 domains, with Cronbach's alpha ranging from 0.867 to 0.928 and McDonald's omega from 0.870 to 0.931 (see Table 6). For the total 20-item scale, α was 0.960 and ω was 0.968. Item-level diagnostics showed acceptable corrected item–total correlations, with no meaningful improvement in either coefficient following item deletion (see supplementary materials Table S9).

**Table 6:** Internal Consistency of the Indonesian PSPSQ 2.0.

Domain	No. of items	Cronbach's α (95% CI)	McDonald's ωt (95% CI)
Quality of Care (QOC)	10	0.928 (0.903–0.946)	0.931 (0.909–0.948)
Interpersonal Relationship (IPR)	6	0.897 (0.867–0.918)	0.901 (0.874–0.922)
Overall (OV)	4	0.867 (0.812–0.903)	0.870 (0.823–0.905)
Total scale	20	0.960 (0.946–0.969)	0.968 (0.959–0.977)

## 4 Discussion

This study provides initial evidence on the translation, cultural adaptation, and initial psychometric evaluation of the Indonesian version of the Patient Satisfaction with Pharmacist Services Questionnaire (PSPSQ 2.0) in community pharmacies and public primary healthcare centres (*Puskesmas*). The availability of a culturally adapted instrument is relevant in Indonesia, where pharmacists are increasingly involved in patient-centered services, including medication counseling, minor ailment management, and chronic disease support.^32^, ^33^ In this context, measuring patient satisfaction may support service evaluation and quality improvement in pharmacist-provided care.^34^

The QOC, IPR, and OV domain scores did not differ significantly between community pharmacies and *Puskesmas*. This additional analysis provided a comparison of observed domain scores across the two practice settings. However, the Mann–Whitney *U* test assesses differences in observed score distributions and does not establish measurement equivalence or demonstrate that the instrument functions similarly across practice settings.^35^ Further studies with sufficiently large samples from each setting should examine measurement invariance using multigroup CFA or differential item functioning analysis.^36^, ^37^

The translation and cultural adaptation process provided initial support for the linguistic and conceptual suitability of the Indonesian PSPSQ 2.0. The experts' assessment supported the relevance of the items, while cognitive debriefing provided additional evidence of their comprehensibility and cultural appropriateness. Minor wording refinements were made while retaining all items, indicating that the adaptation preserved the intended content and meaning of the original instrument. These findings support the use of the Indonesian PSPSQ 2.0 in primary healthcare settings.^38^, 39.

The assessment of sampling adequacy and factorability provides important methodological support for the validity of the extracted structure.^40^ The overall KMO values exceeded recommended thresholds and the significant Bartlett's test indicate that the inter-item correlations were sufficient to justify factor analysis, suggesting that the data structure is appropriate for identifying latent constructs.^40^, ^41^ These findings indicate sufficient shared variance among the items to support identification of the underlying factor structure, consistent with previous validation studies of the PSPSQ 2.0.^42^, ^43^ The scree plot showed an inflection after the third factor, while the Kaiser criterion and Horn's parallel analysis supported retention of three factors. In the parallel analysis, the observed eigenvalues for the first three factors exceeded the corresponding mean resampled eigenvalues. The three-factor solution was also broadly consistent with the conceptual domains of the original PSPSQ 2.0.^10^ Taken together, the statistical evidence and theoretical interpretability supported retention of the three-factor solution. Taken together, these findings provided evidence in favor of a preliminary three-factor solution, which requires further confirmation using confirmatory factor analysis in an independent sample.^44^

The intercorrelation findings suggest that the three factors capture closely related aspects of patient experience while remaining conceptually distinguishable. Although several items showed alternative factor loadings, none met the predefined criterion for substantive cross-loading, supporting generally clear item–domain alignment.^28^ The predominance of the first factor may nevertheless suggest a broader underlying satisfaction dimension. Given the interconnected nature of pharmacist-provided care, this possibility is conceptually plausible but should be interpreted cautiously. Future studies should use CFA to compare the correlated three-factor and bifactor models, with the latter testing whether a general satisfaction factor accounts for common variance beyond the domain-specific factors.^44^

The Indonesian PSPSQ 2.0 demonstrated high internal consistency across all three domains, with comparable Cronbach's alpha and McDonald's omega estimates, supporting the consistency of item responses within each domain.^45^ The high coefficients for the total scale should be interpreted cautiously, as they may partly reflect the number of items and substantial correlations among domains, rather than indicating a unidimensional construct.^46^ Item-deletion analyses showed no meaningful improvement in internal consistency, while item-level indicators provided complementary evidence of item performance.^47^ Reporting confidence intervals for both coefficients further strengthens the interpretation by providing information on the precision of the estimates.^48^

From a practical perspective, the Indonesian PSPSQ 2.0 may provide a structured approach for pharmacists, managers, and researchers to assess patient-reported experience across quality of care, interpersonal relationships, and overall satisfaction. Items addressing communication, respect, trust, and support may help identify areas for service improvement within primary care. Further research is needed to examine how PSPSQ 2.0 scores relate to service characteristics, patient outcomes, and quality improvement interventions.

Several limitations should be acknowledged. First, the study was conducted in community pharmacies and public primary healthcare centres, which may limit the applicability of the findings to hospital or specialized care contexts. Second, the use of self-reported data may introduce response and recall bias, although the six-month recall period was intended to minimise recall difficulties. Third, regional diversity and purposive online sampling may limit generalizability and introduce selection bias, while the cross-sectional design precludes assessment of temporal stability and responsivenes. In addition, the psychometric evaluation was conducted using a combined primary-care sample because the sample sizes within community pharmacies and *Puskesmas* were insufficient for separate factor analyses. Therefore, the preliminary factor structure identified in this study requires further evaluation within both practice settings, particularly using sufficiently samples to assess its stability and measurement equivalence. Fourth, although submission IP addresses and timestamps were used to screen for potential duplicate responses, duplicate participation could not be definitively ruled out because the survey was anonymous and neither IP addresses nor timestamps uniquely identify individual respondents. Finally, this study evaluated content validity, structural validity, and internal consistency but did not assess other measurement properties, including test–retest reliability, confirmatory factor analysis, convergent validity, known-groups validity, measurement invariance, and responsiveness. Accordingly, the three-factor structure and broader psychometric properties of the Indonesian PSPSQ 2.0 remain preliminary and warrant further evaluation in independent, sufficiently large, and diverse samples, including longitudinal studies.

## 5 Conclusion

This study translated and cross-culturally adapted the PSPSQ 2.0 and provides preliminary evidence of its linguistic appropriateness, contextual relevance, and internal consistency for assessing patient satisfaction with pharmacist services in community pharmacies and public primary healthcare centres (*Puskesmas*) in Indonesia. The instrument may provides a standardized approach to assessing patient satisfaction and support service evaluation and quality improvement in these settings. However, the findings should be interpreted cautiously given the study population and online purposive sampling, and the proposed factor structure remains preliminary. Further studies using more diverse and representative samples are needed to confirm the factor structure using CFA, evaluate additional measurement properties, and establish the broader applicability of the instrument across healthcare settings and patient populations.

## CRediT authorship contribution statement

**Irianti Bahana Maulida Reyaan:** Writing – review & editing, Writing – original draft, Methodology, Investigation, Formal analysis, Data curation, Conceptualization. **Tjokorde Istri Armina Padmasawitri:** Writing – review & editing, Validation, Methodology, Formal analysis. **Sherly Meilianti:** Writing – review & editing, Validation, Methodology, Formal analysis. **I Ketut Adnyana:** Writing – review & editing, Validation, Supervision. **Lia Amalia:** Writing – review & editing, Validation, Supervision, Methodology, Conceptualization.

## Funding sources

This study was supported by Indonesia Education Scholarship (Beasiswa Pendidikan Indonesia-BPI), Center for Higher Education Funding and Assessment (Pusat Pembiayaan dan Asesmen Pendidikan Tinggi – PPAPT), the Ministry of Higher Education, Science, and Technology of Republic Indonesia (Kemdiktisaintek), and Indonesian Endowment Fund for Education (Lembaga Pengelola Dana Pendidikan-LPDP) for the research funding [Grant Number: 202404121347].

## Declaration of competing interest

The authors declare that they have no known competing financial interests or personal relationships that could have appeared to influence the work reported in this paper.
