# Supplementary material for: Translation, cross-cultural adaptation and initial psychometric evaluation of the Indonesian PSPSQ 2.0 in community pharmacies and public primary healthcare centres
Source: Explor Res Clin Soc Pharm. 2026 Sep 14;24:100843. doi: 10.1016/j.rcsop.2026.100843 (PMC13595167; doi:10.1016/j.rcsop.2026.100843)
Supplement: Supplementary file 1 [file mmc1.docx]

Table S1: Original Version of PSPSQ 2.0

***Please complete this survey by checking the option that best describes your opinion:***

|  | **Quality of Care** | Strongly Agree | Agree | Disagree | Strongly Disagree |
| --- | --- | --- | --- | --- | --- |
| 1 | The pharmacist fully addressed the main health reason/concerns/issues during my visit. | 4 | 3 | 2 | 1 |
| 2 | The pharmacist was professional in all of our interactions. | 4 | 3 | 2 | 1 |
| 3 | The pharmacist explained information to me in a manner that I could understand. | 4 | 3 | 2 | 1 |
| 4 | The pharmacist checked to see if I understood all the information. | 4 | 3 | 2 | 1 |
| 5 | The pharmacist spent as much time necessary to help me with my questions and concerns. | 4 | 3 | 2 | 1 |
| 6 | The pharmacist made sure I understood how important it is to follow the drug regimen. | 4 | 3 | 2 | 1 |
| 7 | The pharmacist provided useful recommendations on how to take my medications. | 4 | 3 | 2 | 1 |
| 8 | The pharmacist provided useful recommendations about managing my overall health (e.g. diet, exercise). | 4 | 3 | 2 | 1 |
| 9 | The pharmacist worked with me to manage my medication related issues (e.g. cost, side effects of drugs). | 4 | 3 | 2 | 1 |
| 10 | The pharmacist followed up on my progress in a timely manner. | 4 | 3 | 2 | 1 |
|  | | | | | |
|  | **Interpersonal Relationship (pharmacist/patient)** | Strongly Agree | Agree | Disagree | Strongly Disagree |
| 11 | The pharmacist was caring and kind in dealing with my health issues. | 4 | 3 | 2 | 1 |
| 12 | The pharmacist encouraged me to achieve my treatment goals. | 4 | 3 | 2 | 1 |
| 13 | I felt comfortable in my interactions with the pharmacist. | 4 | 3 | 2 | 1 |
| 14 | The pharmacist was respectful to me during our interactions. | 4 | 3 | 2 | 1 |
| 15 | The pharmacist was committed to improving my health. | 4 | 3 | 2 | 1 |
| 16 | I could trust the information that the pharmacist provided. | 4 | 3 | 2 | 1 |
|  | | | | | |
|  | **Overall** | Strongly Agree | Agree | Disagree | Strongly Disagree |
| 17 | I was satisfied with the overall care provided by my pharmacist. | 4 | 3 | 2 | 1 |
| 18 | I would recommend my pharmacist to people I know. | 4 | 3 | 2 | 1 |
| 19 | If needed, I would continue seeing this pharmacist for my healthcare needs. | 4 | 3 | 2 | 1 |

| 20 | The overall care provided by the pharmacist. | Exceeded my expectations  4 | Met my expectations  3 | Did not meet my expectations  2 | Had no expectations  1 |
| --- | --- | --- | --- | --- | --- |

Copyrighted Law and Bounthavong 2007 revised 2014

Table S2 : Characteristics of Experts for Content Validity Assessment

| **No** | **Expert Code** | **Age (years)** | **Gender** | **Years of Practice** | **Area of Work** | **Highest Academic Qualification** |
| --- | --- | --- | --- | --- | --- | --- |
| 1 | E1 | 38 | Female | 12 | Academic | Doctoral (PhD) |
| 2 | E2 | 45 | Female | 20 | Primary Healthcare | Master’s Degree |
| 3 | E3 | 41 | Female | 17 | Community Pharmacy | Master’s Degree |
| 4 | E4 | 35 | Male | 12 | Community Pharmacy | Master’s Degree |
| 5 | E5 | 45 | Female | 19 | Community Pharmacy | Bachelor’s Degree |
| 6 | E6 | 50 | Female | 23 | Policy / Government | Master’s Degree |
| 7 | E7 | 58 | Male | 31 | Academic | Doctoral (PhD) |
| 8 | E8 | 39 | Female | 15 | Primary Healthcare | Master’s Degree |
| 9 | E9 | 44 | Male | 18 | Community Pharmacy | Master’s Degree |
| 10 | E10 | 55 | Female | 30 | Primary Healthcare | Bachelor’s Degree |

Table S3 : Comparison of Component Scores by Practice Setting

| Domain | Community pharmacy  (n=91) | Public primary healthcare centre  (n=109) | p-value* |
| --- | --- | --- | --- |
| QOC | 3.40 (1.10) | 3.50 (0.90) | 0.403 |
| IPR | 3.33 (1.00) | 3.50 (1.00) | 0.806 |
| OV | 3.25 (1.00) | 3.25 (0.75) | 0.716 |

Values are presented as median (IQR). *p*-values were calculated using the two-sided Mann–Whitney U test. Statistical significance was set at *p* < 0.05. QOC: Quality of care; IPR: Interpersonal Relationship (pharmacist/patient); OV: Overall satisfaction

Table S4 : Content Validity Assessment of the Indonesian PSPSQ 2.0 Using a 4-Point Relevance Scale

| Item |  | Expert 1 | Expert 2 | Expert 3 | Expert 4 | Expert 5 | Expert 6 | Expert 7 | Expert 8 | Expert 9 | Expert 10 | I-CVI | S-CVI/Ave | | |
| --- | --- | --- | --- | --- | --- | --- | --- | --- | --- | --- | --- | --- | --- | --- | --- |
| Quality of care | | | | | | | | | | | | | |  |  |
| 1 |  | ✓ | ✓ | ✓ | ✓ | ✓ | ✓ | ✓ | ✓ | ✓ | ✓ | 1.00 |  | | |
| 2 |  | ✓ | - | ✓ | ✓ | ✓ | ✓ | - | ✓ | ✓ | ✓ | 0.80 |  | | |
| 3 |  | ✓ | ✓ | ✓ | ✓ | ✓ | ✓ | ✓ | ✓ | ✓ | ✓ | 1.00 |  | | |
| 4 |  | ✓ | ✓ | ✓ | ✓ | ✓ | ✓ | ✓ | ✓ | ✓ | ✓ | 1.00 | 0.97 | | |
| 5 |  | - | ✓ | ✓ | ✓ | ✓ | ✓ | ✓ | ✓ | ✓ | ✓ | 0.90 |  | | |
| 6 |  | ✓ | ✓ | ✓ | ✓ | ✓ | ✓ | ✓ | ✓ | ✓ | ✓ | 1.00 |  | | |
| 7 |  | ✓ | ✓ | ✓ | ✓ | ✓ | ✓ | ✓ | ✓ | ✓ | ✓ | 1.00 |  | | |
| 8 |  | ✓ | ✓ | ✓ | ✓ | ✓ | ✓ | ✓ | ✓ | ✓ | ✓ | 1.00 |  | | |
| 9 |  | ✓ | ✓ | ✓ | ✓ | ✓ | ✓ | ✓ | ✓ | ✓ | ✓ | 1.00 |  | | |
| 10 |  | ✓ | ✓ | ✓ | ✓ | ✓ | ✓ | ✓ | ✓ | ✓ | ✓ | 1.00 |  | | |
| Interpersonal Relationship | | | | | | | | | | | | | | |  |
| 1 |  | ✓ | ✓ | ✓ | ✓ | ✓ | ✓ | ✓ | ✓ | ✓ | ✓ | 1.00 |  | | |
| 2 |  | ✓ | ✓ | ✓ | ✓ | ✓ | ✓ | ✓ | ✓ | ✓ | ✓ | 1.00 |  | | |
| 3 |  | ✓ | ✓ | ✓ | ✓ | ✓ | ✓ | ✓ | ✓ | ✓ | ✓ | 1.00 |  | | |
| 4 |  | ✓ | ✓ | ✓ | ✓ | ✓ | ✓ | ✓ | - | ✓ | - | 0.80 | 0.97 | | |
| 5 |  | ✓ | ✓ | ✓ | ✓ | ✓ | ✓ | ✓ | ✓ | ✓ | ✓ | 1.00 |  | | |
| 6 |  | ✓ | ✓ | ✓ | ✓ | ✓ | ✓ | ✓ | ✓ | ✓ | ✓ | 1.00 |  | | |
| Overall satisfaction | | | | | | | | | | | | | | |  |
| 1 |  | ✓ | ✓ | ✓ | ✓ | ✓ | ✓ | ✓ | ✓ | ✓ | ✓ | 1.00 |  | | |
| 2 |  | ✓ | ✓ | ✓ | ✓ | ✓ | ✓ | ✓ | ✓ | ✓ | ✓ | 1.00 | 1.00 | | |
| 3 |  | ✓ | ✓ | ✓ | ✓ | ✓ | ✓ | ✓ | ✓ | ✓ | ✓ | 1.00 |  | | |
| 4 |  | ✓ | ✓ | ✓ | ✓ | ✓ | ✓ | ✓ | ✓ | ✓ | ✓ | 1.00 |  | | |
| Overall S-CVI/Ave 0.975 | | | | | | | | | | | | | | | |

Table S5 : Cognitive Debriefing and Revisions of the Indonesian PSPSQ 2.0

| **Item** | **Original Indonesian Version** | **Participants Feedback** | **Revised Indonesian Version** | **Reason for Revision** |
| --- | --- | --- | --- | --- |
| Q2 | Apoteker bersikap profesional dalam semua interaksi kami. | The phrase *"interaksi kami"* sounds less natural in Bahasa Indonesia. | Apoteker bersikap profesional dalam berinteraksi dengan saya. | To improve clarity and natural language use while preserving the original meaning. |
| Q5 | Apoteker memberikan waktu yang diperlukan untuk membantu saya bertanya dan menjelaskan kekuatiran saya. | The wording is lengthy and less conversational. | Apoteker meluangkan waktu untuk mendengarkan pertanyaan dan kekuatiran saya. | To enhance readability and better reflect patient-centered communication. |
| Q14 | Apoteker bersikap hormat kepada saya selama interaksi kami. | Clarification was requested regarding the interpretation of "respectful" in the Indonesian cultural context. | No wording change; explanatory guidance was provided during expert review. | The term was considered conceptually equivalent and understandable after clarification. |

Table S6 : Forward-backward translation and pilot testing results

| No. | Original Questionnaire | Forward Translation | Backward Translation | Pilot Testing/Face Validity |
| --- | --- | --- | --- | --- |
| **1.** | **Quality of Care** | **Kualitas Pelayanan Kesehatan** | **Quality of Healthcare** | **Kualitas Pelayanan Kesehatan** |
|  | 1. The pharmacist fully addressed the main health reason/concerns/issues during my visit. | 1. Apoteker memberikan perhatian penuh atas alasan/kekuatiran/masalah kesehatan utama saya selama kunjungan saya. | 1. The pharmacist gave full attention to my main health reasons/concerns/issues during my visit. | 1. Apoteker memberikan perhatian penuh atas alasan/kekuatiran/masalah kesehatan utama saya selama kunjungan saya. |
|  | 2. The pharmacist was professional in all of our interactions. | 2. Apoteker bersikap profesional dalam semua interaksi kami. | 2. The pharmacist was professional in all of our interactions. | 2. Apoteker bersikap profesional dalam berinteraksi dengan saya. |
|  | 3. The pharmacist explained information to me in a manner that I could understand. | 3. Apoteker menjelaskan informasi kepada saya dengan cara yang dapat saya pahami. | 3. The pharmacist explained information to me in a way I could understand. | 3. Apoteker menjelaskan informasi kepada saya dengan cara yang dapat saya pahami. |
|  | 4. The pharmacist checked to see if I understood all the information. | 4. Apoteker memastikan saya memahami semua informasi yang dijelaskan. | 4. The pharmacist made sure that I understood all information explained. | 4. Apoteker memastikan saya memahami semua informasi yang dijelaskan. |
|  | 5. The pharmacist spent as much time necessary to help me with my questions and concerns. | 5. Apoteker memberikan waktu yang diperlukan untuk membantu saya bertanya dan menjelaskan kekuatiran saya. | 5. The pharmacist gave time required to assist me with my questions and explain my concerns. | 5. Apoteker meluangkan waktu untuk mendengarkan pertanyaan dan kekuatiran saya. |
|  | 6. The pharmacist made sure I understood how important it is to follow the drug regimen. | 6. Apoteker memastikan saya memahami betapa pentingnya mengikuti regimen pengobatan. | 6. The pharmacist made sure that I understood how important it is to follow the medication regimen. | 6. Apoteker memastikan saya memahami betapa pentingnya mengikuti regimen pengobatan. |
|  | 7. The pharmacist provided useful recommendations on how to take my medications. | 7. Apoteker memberikan rekomendasi yang bermanfaat tentang cara mengonsumsi obat-obatan saya. | 7. The pharmacist gave useful recommendations on how to take my medicine. | 7. Apoteker memberikan rekomendasi yang bermanfaat tentang cara mengonsumsi obat-obatan saya. |
|  | 8. The pharmacist provided useful recommendations about managing my overall health (e.g. diet, exercise). | 8. Apoteker memberikan rekomendasi yang bermanfaat tentang pengelolaan kesehatan saya secara menyeluruh (misalnya, diet, olahraga). | 8. The pharmacist gave useful recommendations on the management of my overall health (such as, diet, exercise). | 8. Apoteker memberikan rekomendasi yang bermanfaat tentang pengelolaan kesehatan saya secara menyeluruh (misalnya, diet, olahraga). |
|  | 9. The pharmacist worked with me to manage my medication related issues (e.g. cost, side effects of drugs). | 9. Apoteker bekerjasama dengan saya untuk mengelola masalah terkait pengobatan saya (misalnya, biaya, efek samping obat). | 9. The pharmacist cooperated with me to manage issues related to my medication (such as, costs, side effects of medicine). | 9. Apoteker bekerjasama dengan saya untuk mengelola masalah terkait pengobatan saya (misalnya, biaya, efek samping obat). |
|  | 10. The pharmacist followed up on my progress in a timely manner. | 10. Apoteker menindaklanjuti perkembangan kesehatan saya secara tepat waktu. | 10. The pharmacist followed up on my health progress in a timely manner. | 10. Apoteker menindaklanjuti perkembangan kesehatan saya secara tepat waktu. |
| 2. | **Interpersonal Relationship (pharmacist/patient)** | **Hubungan Antarpribadi (apoteker/pasien** | **Interpersonal Relationship (pharmacist/patient)** | **Hubungan Antarpribadi (apoteker/pasien** |
|  | 11. The pharmacist was caring and kind in dealing with my health issues. | 11. Apoteker peduli dan ramah dalam menangani masalah kesehatan saya. | 11. The pharmacist was caring and kind in dealing with my health issues. | 11. Apoteker peduli dan ramah dalam menangani masalah kesehatan saya. |
|  | 12. The pharmacist encouraged me to achieve my treatment goals. | 12. Apoteker menyemangati saya untuk mencapai tujuan pengobatan saya. | 12. The pharmacist encouraged me to achieve the purpose of my medication. | 12. Apoteker menyemangati saya untuk mencapai tujuan pengobatan saya. |
|  | 13. I felt comfortable in my interactions with the pharmacist. | 13. Saya merasa nyaman selama saya berinteraksi dengan apoteker. | 13. I felt comfortable when interacting with the pharmacist. | 13. Saya merasa nyaman selama saya berinteraksi dengan apoteker. |
|  | 14. The pharmacist was respectful to me during our interactions. | 14. Apoteker bersikap hormat kepada saya selama interaksi kami. | 14. The pharmacist was respectful to me during our interactions. | 14. Apoteker bersikap hormat kepada saya selama interaksi kami. |
|  | 15. The pharmacist was committed to improving my health. | 15. Apoteker berkomitmen untuk meningkatkan kesehatan saya. | 15. The pharmacist was committed to improving my health. | 15. Apoteker berkomitmen untuk meningkatkan kesehatan saya. |
|  | 16. I could trust the information that the pharmacist provided. | 16. Saya dapat mempercayai informasi yang diberikan oleh apoteker. | 16. I could trust the information given by the pharmacist. | 16. Saya dapat mempercayai informasi yang diberikan oleh apoteker. |
| **3.** | **Overall** | **Keseluruhan** | **Overall** | **Keseluruhan** |
|  | 17. I was satisfied with the overall care provided by my pharmacist. | 17. Saya merasa puas dengan seluruh pelayanan kesehatan yang diberikan oleh apoteker | 17. I was satisfied with the overall healthcare provided by my pharmacist. | 17. Saya merasa puas dengan seluruh pelayanan kesehatan yang diberikan oleh apoteker |
|  | 18. I would recommend my pharmacist to people I know. | 18. Saya akan merekomendasikan apoteker saya kepada orang-orang yang saya kenal. | 18. I would recommend my pharmacist to people I know. | 18. Saya akan merekomendasikan apoteker saya kepada orang-orang yang saya kenal. |
|  | 19. If needed, I would continue seeing this pharmacist for my healthcare needs. | 19. Jika diperlukan, saya akan terus mengunjungi apoteker saya untuk kebutuhan perawatan kesehatan saya. | 19. If required, I would continue visiting my pharmacist for my healthcare needs. | 19. Jika diperlukan, saya akan terus mengunjungi apoteker saya untuk kebutuhan perawatan kesehatan saya. |
|  | 20. The overall care provided by the pharmacist. | 20. Pelayanan kesehatan menyeluruh oleh apoteker. | 20. The overall healthcare by the pharmacist. | 20. Pelayanan kesehatan menyeluruh oleh apoteker. |

Table S7: Correlation Matrix

| **Item** | **QOC1** | **QOC2** | **QOC3** | **QOC4** | **QOC5** | **QOC6** | **QOC7** | **QOC8** | **QOC9** | **QOC10** | **IPR1** | **IPR2** | **IPR3** | **IPR4** | **IPR5** | **IPR6** | **OV1** | **OV2** | **OV3** | **OV4** |
| --- | --- | --- | --- | --- | --- | --- | --- | --- | --- | --- | --- | --- | --- | --- | --- | --- | --- | --- | --- | --- |
| **QOC1** | 1.000 | 0.562 | 0.601 | 0.673 | 0.630 | 0.703 | 0.711 | 0.621 | 0.604 | 0.659 | 0.706 | 0.571 | 0.544 | 0.669 | 0.583 | 0.573 | 0.650 | 0.583 | 0.619 | 0.621 |
| **QOC2** | 0.562 | 1.000 | 0.604 | 0.582 | 0.596 | 0.590 | 0.638 | 0.527 | 0.479 | 0.486 | 0.575 | 0.473 | 0.541 | 0.519 | 0.390 | 0.599 | 0.656 | 0.460 | 0.616 | 0.464 |
| **QOC3** | 0.601 | 0.604 | 1.000 | 0.668 | 0.611 | 0.611 | 0.676 | 0.507 | 0.521 | 0.439 | 0.423 | 0.459 | 0.394 | 0.474 | 0.409 | 0.493 | 0.527 | 0.412 | 0.408 | 0.469 |
| **QOC4** | 0.673 | 0.582 | 0.668 | 1.000 | 0.730 | 0.739 | 0.731 | 0.617 | 0.612 | 0.610 | 0.556 | 0.564 | 0.487 | 0.542 | 0.525 | 0.563 | 0.573 | 0.647 | 0.553 | 0.542 |
| **QOC5** | 0.630 | 0.596 | 0.611 | 0.730 | 1.000 | 0.739 | 0.715 | 0.628 | 0.623 | 0.554 | 0.687 | 0.599 | 0.550 | 0.637 | 0.508 | 0.608 | 0.629 | 0.623 | 0.566 | 0.514 |
| **QOC6** | 0.703 | 0.590 | 0.611 | 0.739 | 0.739 | 1.000 | 0.824 | 0.670 | 0.698 | 0.614 | 0.666 | 0.584 | 0.500 | 0.607 | 0.557 | 0.580 | 0.586 | 0.556 | 0.591 | 0.499 |
| **QOC7** | 0.711 | 0.638 | 0.676 | 0.731 | 0.715 | 0.824 | 1.000 | 0.693 | 0.676 | 0.661 | 0.650 | 0.626 | 0.574 | 0.718 | 0.624 | 0.584 | 0.636 | 0.620 | 0.570 | 0.537 |
| **QOC8** | 0.621 | 0.527 | 0.507 | 0.617 | 0.628 | 0.670 | 0.693 | 1.000 | 0.839 | 0.746 | 0.583 | 0.695 | 0.525 | 0.510 | 0.584 | 0.419 | 0.546 | 0.631 | 0.638 | 0.528 |
| **QOC9** | 0.604 | 0.479 | 0.521 | 0.612 | 0.623 | 0.698 | 0.676 | 0.839 | 1.000 | 0.750 | 0.558 | 0.536 | 0.482 | 0.514 | 0.569 | 0.434 | 0.565 | 0.603 | 0.513 | 0.491 |
| **QOC10** | 0.659 | 0.486 | 0.439 | 0.610 | 0.554 | 0.614 | 0.661 | 0.746 | 0.750 | 1.000 | 0.626 | 0.688 | 0.566 | 0.565 | 0.660 | 0.500 | 0.587 | 0.660 | 0.587 | 0.537 |
| **IPR1** | 0.706 | 0.575 | 0.423 | 0.556 | 0.687 | 0.666 | 0.650 | 0.583 | 0.558 | 0.626 | 1.000 | 0.647 | 0.650 | 0.727 | 0.604 | 0.621 | 0.689 | 0.571 | 0.691 | 0.611 |
| **IPR2** | 0.571 | 0.473 | 0.459 | 0.564 | 0.599 | 0.584 | 0.626 | 0.695 | 0.536 | 0.688 | 0.647 | 1.000 | 0.658 | 0.648 | 0.720 | 0.541 | 0.656 | 0.460 | 0.616 | 0.464 |
| **IPR3** | 0.544 | 0.541 | 0.394 | 0.487 | 0.550 | 0.500 | 0.574 | 0.525 | 0.482 | 0.566 | 0.650 | 0.658 | 1.000 | 0.793 | 0.604 | 0.637 | 0.527 | 0.412 | 0.408 | 0.469 |
| **IPR4** | 0.669 | 0.519 | 0.474 | 0.542 | 0.637 | 0.607 | 0.718 | 0.510 | 0.514 | 0.565 | 0.727 | 0.648 | 0.793 | 1.000 | 0.658 | 0.554 | 0.573 | 0.647 | 0.553 | 0.542 |
| **IPR5** | 0.583 | 0.390 | 0.409 | 0.525 | 0.508 | 0.557 | 0.624 | 0.584 | 0.569 | 0.660 | 0.604 | 0.720 | 0.604 | 0.658 | 1.000 | 0.484 | 0.507 | 0.609 | 0.535 | 0.530 |
| **IPR6** | 0.573 | 0.599 | 0.493 | 0.563 | 0.608 | 0.580 | 0.584 | 0.419 | 0.434 | 0.500 | 0.621 | 0.541 | 0.637 | 0.554 | 0.484 | 1.000 | 0.711 | 0.556 | 0.683 | 0.455 |
| **OV1** | 0.650 | 0.656 | 0.527 | 0.573 | 0.629 | 0.586 | 0.636 | 0.546 | 0.565 | 0.587 | 0.689 | 0.656 | 0.527 | 0.573 | 0.507 | 0.711 | 1.000 | 0.706 | 0.677 | 0.644 |
| **OV2** | 0.583 | 0.460 | 0.412 | 0.647 | 0.623 | 0.556 | 0.620 | 0.631 | 0.603 | 0.660 | 0.644 | 0.610 | 0.715 | 0.651 | 0.609 | 0.556 | 0.706 | 1.000 | 0.704 | 0.631 |
| **OV3** | 0.619 | 0.616 | 0.408 | 0.553 | 0.566 | 0.591 | 0.570 | 0.638 | 0.513 | 0.587 | 0.605 | 0.642 | 0.595 | 0.531 | 0.535 | 0.683 | 0.677 | 0.704 | 1.000 | 0.568 |
| **OV4** | 0.621 | 0.464 | 0.469 | 0.464 | 0.514 | 0.499 | 0.537 | 0.528 | 0.491 | 0.537 | 0.500 | 0.532 | 0.462 | 0.493 | 0.530 | 0.455 | 0.644 | 0.631 | 0.568 | 1.000 |

Table S8: Comparison of One-, Two-, and Three-Factor Solutions in Exploratory Factor Analysis

| **Criterion** | **1-factor solution** |  | **2-factor solution** | **3-factor solution** |
| --- | --- | --- | --- | --- |
| **Scree plot** | Not supported |  | Not supported | Supported |
| **Kaiser criterion** | Not supported |  | Not supported | 3 factors indicated |
| **Horn’s parallel analysis** | Not supported |  | Not supported | 3 factors indicated |
| **χ²** | 970.548 |  | 753.057 | 562.470 |
| **df** | 170 |  | 151 | 133 |
| **χ²/df** | 5.71 |  | 4.99 | 4.23 |
| **RMSEA** | 0.153 |  | 0.121 | 0.075 |
| **TLI** | 0.753 |  | 0.790 | 0.909 |
| **BIC** | 69.835 |  | −46.989 | −142.206 |
| **Overall assessment** | Not retained |  | Not retained | Retained preliminarily |

Figure S1. Scree plot of the observed eigenvalues of the Indonesian PSPSQ 2.0

Figure S2. Horn’s parallel analysis for determining the number of factors in the Indonesian PSPSQ 2.0.

| **Factor** | **Observed eigenvalue** | **Mean simulated eigenvalue** | **Mean resampled eigenvalue** | **Factor-retention interpretation** |
| --- | --- | --- | --- | --- |
| 1 | 12.031 | 0.708 | 1.002 | Retain |
| 2 | 0.946 | 0.589 | 0.840 | Retain |
| 3 | 0.810 | 0.508 | 0.731 | Retain |
| 4 | 0.512 | 0.431 | 0.628 | Do not retain |
| 5 | 0.293 | 0.364 | 0.534 | Do not retain |
| 6 | 0.261 | 0.302 | 0.454 | Do not retain |

The figure compares observed eigenvalues with mean resampled eigenvalues, with the Kaiser criterion (eigenvalue >1) shown for reference. The accompanying table presents the observed, mean simulated, and mean resampled eigenvalues and the corresponding factor-retention interpretation. The parallel analysis supported retention of three factors.

Table S9: Item-Level Analysis of Internal Consistency for the Indonesian PSPSQ 2.0

| **Domain** | **Item** | **Scale mean if item deleted** |  | **Scale variance if item deleted** | **Corrected item–total correlation** | **Squared multiple correlation** | **Cronbach’s α if item deleted** | **McDonald’s ω if item deleted** |
| --- | --- | --- | --- | --- | --- | --- | --- | --- |
| Quality of Care (QOC) | QOC1 | 30.870 |  | 23.330 | 0.696 | 0.506 | 0.921 | 0.922 |
|  | QOC2 | 30.750 |  | 23.907 | 0.693 | 0.539 | 0.922 | 0.922 |
|  | QOC3 | 30.715 |  | 23.541 | 0.746 | 0.665 | 0.920 | 0.921 |
|  | QOC4 | 30.815 |  | 22.935 | 0.763 | 0.662 | 0.918 | 0.919 |
|  | QOC5 | 30.840 |  | 23.210 | 0.704 | 0.553 | 0.921 | 0.922 |
|  | QOC6 | 30.910 |  | 22.163 | 0.760 | 0.621 | 0.918 | 0.918 |
|  | QOC7 | 30.740 |  | 23.198 | 0.755 | 0.605 | 0.919 | 0.919 |
|  | QOC8 | 31.050 |  | 22.028 | 0.717 | 0.592 | 0.921 | 0.921 |
|  | QOC9 | 30.990 |  | 22.191 | 0.740 | 0.621 | 0.919 | 0.919 |
|  | QOC10 | 31.110 |  | 21.907 | 0.696 | 0.571 | 0.923 | 0.923 |
| Domain coefficient | 10 items | — |  | — | — | — | 0.928 | 0.931 |
| Interpersonal Relationship (IPR) | IPR1 | 17.145 |  | 7.089 | 0.800 | 0.661 | 0.867 | 0.871 |
|  | IPR2 | 17.290 |  | 6.709 | 0.709 | 0.552 | 0.883 | 0.885 |
|  | IPR3 | 17.130 |  | 7.330 | 0.725 | 0.542 | 0.878 | 0.882 |
|  | IPR4 | 17.120 |  | 7.242 | 0.742 | 0.581 | 0.876 | 0.878 |
|  | IPR5 | 17.230 |  | 6.681 | 0.744 | 0.586 | 0.876 | 0.878 |
|  | IPR6 | 16.985 |  | 7.834 | 0.647 | 0.486 | 0.890 | 0.891 |
| Domain coefficient | 6 items | — |  | — | — | — | 0.897 | 0.901 |
| Overall (OV) | OV1 | 10.055 |  | 2.695 | 0.746 | 0.571 | 0.820 | 0.824 |
|  | OV2 | 10.110 |  | 2.500 | 0.761 | 0.594 | 0.811 | 0.814 |
|  | OV3 | 10.105 |  | 2.507 | 0.742 | 0.561 | 0.819 | 0.823 |
|  | OV4 | 10.275 |  | 2.713 | 0.628 | 0.394 | 0.866 | 0.867 |
| Domain coefficient | 4 items | — |  | — | — | — | 0.867 | 0.870 |
